# Structural Parameters and Behavior in Simulated Body Fluid of High Entropy Alloy Thin Films

**DOI:** 10.3390/ma17051162

**Published:** 2024-03-01

**Authors:** Doina Craciun, Edwin A. Laszlo, Julia C. Mirza-Rosca, Gabriela Dorcioman, Victor Geanta, Ionelia Voiculescu, Gabriel Craciun, Liviu Badea, Valentin Craciun

**Affiliations:** 1 National Institute for Laser, Plasma and Radiation Physics, 077125 Măgurele, Romania; doina.craciun@inflpr.ro (D.C.); gabriela.dorcioman@inflpr.ro (G.D.); 2 Faculty of Physics, Doctoral School of Physics, University of Bucharest, 077125 Măgurele, Romanialiviu9badea@gmail.com (L.B.); 3 Mechanical Engineering Department, University of Las Palmas de Gran Canaria, 35017 Las Palmas de Gran Canaria, Spain; 4 Materials Engineering and Welding Department, Transilvania University of Brasov, 500036 Brasov, Romania; 5 Faculty of Material Science and Engineering, National University of Science and Technology Politehnica of Bucharest, 060042 Bucharest, Romania; 6 Institute for Microtechnology, 077190 Voluntari, Romania; 7 National R&D Institute for Non-Ferrous and Rare Metals, 077145 Pantelimon, Romania; 8 Extreme Light Infrastructure for Nuclear Physics, IFIN-HH, 077125 Măgurele, Romania

**Keywords:** high-entropy alloy (HEA), high-entropy nitride alloy (HEN), thin films, pulsed laser deposition, electrochemical and corrosion behavior, OCP, EIS, Ringer solution, chemical and physical barrier

## Abstract

The structure, composition and corrosion properties of thin films synthesized using the Pulsed Laser Deposition (PLD) technique starting from a three high entropy alloy (HEA) AlCoCrFeNix produced by vacuum arc remelting (VAR) method were investigated. The depositions were performed at room temperature on Si and mirror-like polished Ti substrates either under residual vacuum (low 10^−7^ mbar, films denoted HEA2, HEA6, and HEA10, which were grown from targets with Ni concentration molar ratio, x, equal to 0.4, 1.2, and 2.0, respectively) or under N_2_ (10^−4^ mbar, films denoted HEN2, HEN6, and HEN10 for the same Ni concentration molar ratios). The deposited films’ structures, investigated using Grazing Incidence X-ray Diffraction, showed the presence of face-centered cubic and body-centered cubic phases, while their surface morphology, investigated using scanning electron microscopy, exhibited a smooth surface with micrometer size droplets. The mass density and thickness were obtained from simulations of acquired X-ray reflectivity curves. The films’ elemental composition, estimated using the energy dispersion X-ray spectroscopy, was quite close to that of the targets used. X-ray Photoelectron Spectroscopy investigation showed that films deposited under a N_2_ atmosphere contained several percentages of N atoms in metallic nitride compounds. The electrochemical behavior of films under simulated body fluid (SBF) conditions was investigated by Open Circuit Potential (OCP) and Electrochemical Impedance Spectroscopy measurements. The measured OCP values increased over time, implying that a passive layer was formed on the surface of the films. It was observed that all films started to passivate in SBF solution, with the HEN6 film exhibiting the highest increase. The highest repassivation potential was exhibited by the same film, implying that it had the highest stability range of all analyzed films. Impedance measurements indicated high corrosion resistance values for HEA2, HEA6, and HEN6 samples. Much lower resistances were found for HEN10 and HEN2. Overall, HEN6 films exhibited the best corrosion behavior among the investigated films. It was noticed that for 24 h of immersion in SBF solution, this film was also a physical barrier to the corrosion process, not only a chemical one.

## 1. Introduction

High-entropy alloys (HEAs) and their nitrides (HENs) are recently discovered structural [1] and functional [2] materials that are already involved in the development of many new and important high-tech applications [3,4] due to their outstanding properties such as hardness and strength [5,6], fatigue resistance [7,8], fracture toughness [9], high-temperature oxidation resistance [10,11], corrosion resistance [12,13], and electrical and magnetic properties [14,15]. These HEA properties have been attributed to four effects: (a) the high entropy, offering the thermodynamics for stabilizing the simple structure; (b) the sluggish diffusion effect, which slows the growth of second phase nuclei out of a single-phase solid solution; (c) the severe lattice distortion due to the presence of atoms having different radii, which provides excess strength and contributes to the slow kinetics in HEAs; and (d) the cocktail effect, which could synergistically enhance the properties by alloying [1,2].

As a result of their particularly high mechanical properties (hardness, ballistic impact, wear) [16,17] and good behavior in chemical environments, high-entropy alloys from the AlCoCrFeNi system have been among the most studied metallic materials in recent years [18,19,20,21]. The microstructures of alloys largely depend on the proportion of alloying elements. Thus, chemical elements such as Al, Cr, and Fe are bcc phase formers, while Ni, Co, and N are fcc stabilizing elements.

Many high-tech applications in the nuclear industry, photocatalysis, or corrosion protection [3,4,12,13,22,23,24,25,26] require the use of thin films, which also have cost benefits, since some elements used in HEA are rather expensive. HEA and HEN thin films were deposited by magnetron sputtering [22] or pulsed laser deposition (PLD) [23]. Recently, we described the deposition of thin HEA and HEN films on Si substrates using the PLD technique starting from AlCoCrFeNi_x_ alloys with a nominal Ni molar content, x, of 0.4, 1.2, and 2.0, respectively [27]. The PLD technique has the advantage that it requires small area targets and the optimization of the growth process is rather simple. Elemental compositional analysis performed using Energy Dispersive X-ray Spectroscopy (EDS) in a Scanning Electron Microscope (SEM) confirmed that the PLD technique provided a good stoichiometric transfer from the target to thin film. The bulk alloys used for targets were obtained by the Vacuum Arc Remelting (VAR) method, varying the nickel content upon alloying from high purity raw materials. More details about the VAR method and bulk HEAs properties can be found in refs. [28,29]. After the synthesis process, the elaborated alloys were used as targets for thin films’ growth using the PLD technique. The structure, chemical elemental composition, surface, and electrochemical and mechanical properties of thin films deposited on Ti substrates were analyzed to understand the effect of composition on the crystalline structure and their corrosion resistance. These studies are required if one wants to explore the use of HEA and HEN thin films as protective coatings for bio-medical applications.

## 2. Experimental Details

### 2.1. Bulk Solid-Solutions Manufacturing

**Figure 1:**
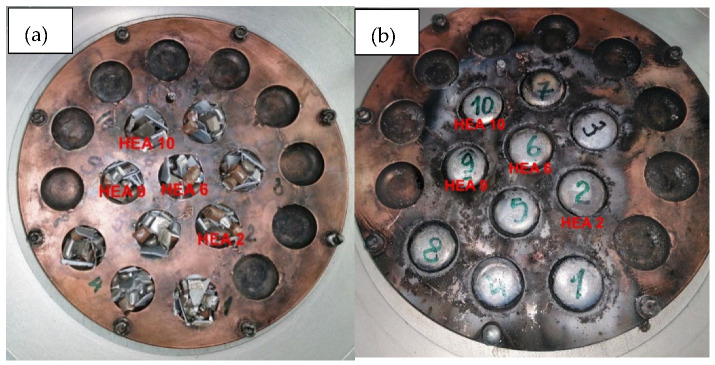
Image of the melting copper plate inside the VAR equipment: (**a**) raw material placed on individual sockets; (**b**) HEA alloy samples after melting.

The HEA mini-ingots that were used as targets were obtained with the VAR method [28,29] using MRF ABJ 900 equipment (Allenstown, Merrimack, NH, USA) in the ERAMET Laboratory, National University of Science and Technology Politechnica Bucharest—Materials Science and Engineering Faculty. High purity granular raw materials, classified according to ASTM B214-16 [30], were used to obtain the metal batches, as shown in Figure 1. For each batch, the masses of raw materials and the mass of the molten parts were weighed using a high precision weighing balance, and then the efficiency of the metallurgical process was calculated. All measured and calculated values are presented in Table 1, where T at the end of a label means target and 2, 6, and 10 refer to AlCoCrFeNix alloys with a nominal Ni molar content x of 0.4, 1.2, and 2.0, respectively. Material losses were low because the working chamber was vacuumed four times before the melting process, and the metallic bath was protected using high-purity (99.999%) Ar flow at 1.2 bar. Melting and remelting operations were performed at least 5 times on each side of the mini-ingots, which were obtained in the form of round buttons of about 32 mm in diameter and 9 mm in thickness (see Figure 1).

The X-ray diffraction (XRD) patterns acquired from the prepared targets showed that for low Ni concentrations, the structure corresponded to a simple body-centered cubic (bcc) one. Increasing the Ni concentration, a second phase of face-centered cubic (fcc) material appeared, while the bcc phase content decreased. For the highest Ni concentration used in this study (x = 2.0), the structure was simple fcc [27]. Ni has a high valence electron concentration (VEC) value and its concentration in an HEA material will greatly influence the overall VEC value. For low Ni concentration, the HEA will have a low VEC value and tends to crystallize in a bcc lattice, while for a high Ni concentration, the HEA material will also have a high VEC value and will crystallize in an fcc lattice. For intermediate values, the HEA will exhibit a mixture of bcc and fcc phases, as was observed in our previous study [27] and reported in the literature [24]. Three alloys with nickel molar fraction x equal to 0.4, 1.2, and 2.0, and with pure bcc, mixed, and pure fcc phases, respectively, were chosen as targets to grow the thin films on Si and Ti substrates by PLD.

### 2.2. Synthesis of HEA and HEN Thin Films

Thin films were grown using the PLD technique at room temperature on silicon substrates, for physical and chemical characterization, and on highly polished, high-purity Ti disks for electrochemical characterization, where the substrate should be a good electrical conductor. Polishing creates a smooth and uniform surface on the substrate, which helps in obtaining consistent and reproducible electrochemical data. Irregularities or roughness on the substrate surface can lead to variations in electrode performance and measurement results. Also, polishing helps to minimize background noise or signals arising from surface irregularities or impurities, thereby improving the signal-to-noise ratio and the overall accuracy of the measurements. Overall, using a polished substrate enhances the reliability, sensitivity, and reproducibility of electrochemical measurements. Ti was also chosen since it is the most frequently used material for biomedical application due to its excellent corrosion resistance properties and biocompatibility. We previously showed that the electrochemical properties of Ti could be improved by using various materials as coatings [31,32]. The substrates were sonicated for 5 min in acetone, alcohol, then in DI water and finally blown dry with high-purity nitrogen just before being loaded into the deposition chamber. High vacuum conditions were reached by using a turbomolecular pump backed by a mechanical pump and a liquid nitrogen cold trap. The HEA targets were firstly mechanically polished and then preablated for 2 min to ensure the same target conditions for all deposited films. The ablation time was varied from 2 to 12 min using a KrF laser (wavelength, 248 nm, pulse duration, ~25 ns) at a fluence of 3 J/cm^2^ and a repetition rate of 40 Hz. The target–substrate distance was set at 5 cm. The depositions were performed under residual vacuum (low 10^−7^ mbar, films denoted as HEA2, HEA6, and HEA10 corresponding to Ni molar ratio, x, equal to 0.4, 1.2, and 2.0, respectively) or N_2_ atmosphere (1 × 10^−4^ mbar, films denoted as HEN2, HEN6, and HEN10 for the same Ni molar ratios).

### 2.3. Sample Characterization

The structure of the deposited films was investigated by Grazing Incidence X-ray Diffraction (GIXRD) with a diffractometer (Empyrean, Malvern, UK) set up to work at a parallel beam geometry. The same instrument was used to collect X-ray reflectivity (XRR) curves in the 0.2°–4.0° 2θ range, which were afterwards simulated using the software package Reflectivity version 1.3a from Malvern. The surface morphology and chemical composition were investigated by scanning electron microscopy (SEM) (Nova NanoSEM 630 with an Element EDS attachment, Eindhoven, The Netherlands). The films’ surface chemical composition was also investigated using X-ray Photoelectron Spectroscopy (XPS) with an ESCALB Xi+ working with monochromatic AlK_α_ radiation. Survey scans were initially acquired, followed by high resolution scans for core level regions of Ni, Fe, Cr, Al, Co, O, C, and N. The binding energy values were referenced to the adventitious C1s position at 284.8 eV. Mechanical properties were measured using a Bruker Triboindentor T980 (Aachen, Germany) equipped with a Berkovich three-sided pyramidal diamond tip, 0.1 mN maximum applied load, loading and unloading sequences of 20 s, and a 5 s dwell time to minimize the creep effect.

All electrochemical measurements for films deposited on polished Ti substrates were carried out in simulated body fluid (SBF), which in our case was Grifols Ringer’s solution from Laboratorios Grifols, Barcelona, Spain. It has the following composition in mmol/l: Na^+^ 129.8; K^+^ 5.6; Ca^2+^ 1.9; Cl^−^ 111.8; and C_3_H_5_O_3_^−^ 27.4. This is a modified physiological solution, partially replacing Na^+^ with Ca^2+^ and K^+^ and some Cl^-^ with C_3_H_5_O^3-^. The lactate ions are transformed into bicarbonate ions, allowing the pH of the solution to be controlled. Electrochemical determinations were performed at 25 °C using a rectangular cell that contained 250 mL electrolyte. The working electrode (the sample to be analyzed) potential was recorded against a KCl (saturated) calomel reference electrode (SSCE) and a Pt cylindrical grid acted as a counter electrode. For Open Circuit Potential (OCP) determinations, the working electrodes were kept in solution for 24 h. After this, for pitting behavior and the measurement of repassivation potential, the potentiodynamic polarization curve was recorded, beginning at an initial potential OCP—200 mV and a scanning rate of 0.1 mV s^−1^. Scanning was stopped once the current density achieved 150 µA cm^−2^. Using a PAR 263 A potentiostat that was paired with a PAR 5210 lock-in amplifier, data on AC impedance were acquired at a variety of potentials. A single sine wave recording was carried out for each specimen at frequencies ranging from 10^−1^ to 10^5^ Hz, and the amplitude of the AC potential was set to 10 millivolts throughout the experiment and ZSimpWin 3.22 software was used to analyze the impedance spectra [33,34].

## 3. Results and Discussion

**Figure 2:**
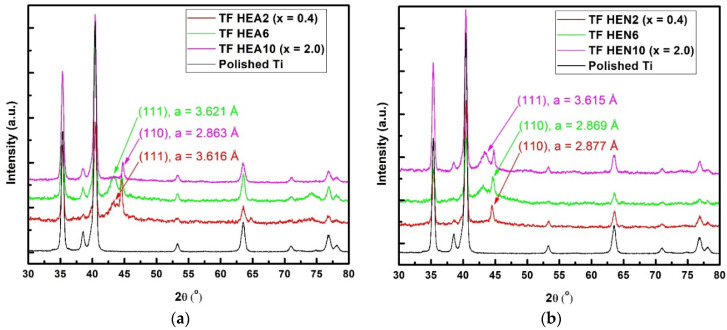
GIXRD diffraction patterns acquired from (**a**) thin HEA films (Ni molar ratio x = 0.4, 1.2, and 2.0) and (**b**) thin HEN films (Ni ratio x = 0.4, 1.2, and 2.0); the XRD patterns of the Ti substrate are also displayed.

The GIXRD patterns acquired from a bare Ti substrate and films deposited on Ti under high vacuum or N_2_ atmosphere from the three different targets used in this study are displayed in Figure 2. The GIXRD patterns acquired from HEN2 and HEA10 films exhibited only the peaks corresponding to the bcc phase, while the rest of the films exhibited peaks from both the fcc and bcc phases. It seems that during the high nonequilibrium deposition process characteristic for PLD, the Ni concentration no longer plays the most important role in deciding which crystalline phase the film will adopt. The lattice parameters of the bcc phases were quite close to the lattice parameter of pure bcc Fe (a = 2.8665 Å), while the lattice parameters of the fcc phases were close to those of pure Ni (a = 3.5245 Å). The main diffraction peaks’ positions and grain sizes estimated using the HighScorePlus 4.1 software from Malvern are displayed in Table 2. It was found that for all deposited films the bcc phase had larger crystallites than the fcc phase. Also, the HEN6 film exhibited smaller crystallites and a larger lattice parameter for the fcc phase, while the bcc phase exhibited a lattice parameter smaller than that of the target used.

**Figure 3:**
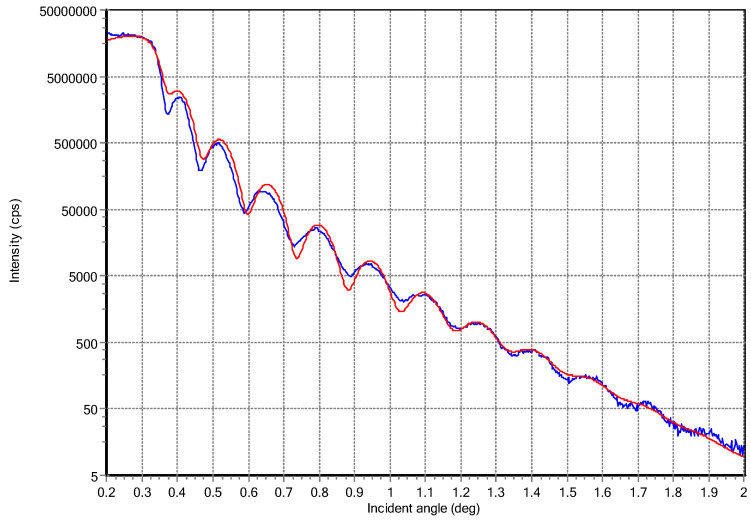
Typical XRR curve (blue trace) acquired from a very thin HEA2 film and its simulation (red trace).

**Figure 4:**
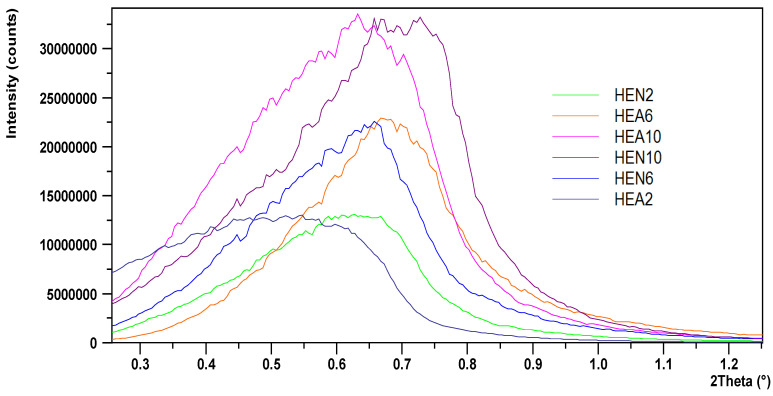
The critical angle region for the acquired XRR curves from HEA and HEN thin films.

A typical XRR curve intensity acquired at very low incidence angles from a very thin HEA2 film, deposited for 2 min only, and its simulation obtained using the software package Reflectivity version 1.3a, are displayed in Figure 3. The simulation results indicated a density of 5.9 g/cm^3^, a surface roughness of around 1 nm, and a thickness of 25.7 nm, which gives a deposition rate of 0.0054 nm/pulse. Films thicker than 125 nm that were used for electrochemical measurements exhibited rougher surface morphologies and did not show oscillations to estimate their thickness based on XRR measurements. The region of the critical angle, which is proportional to the film’s density, for the acquired XRR curves from the deposited films, is displayed in Figure 4. The curves were vertically shifted for a better view of the differences. One can note an increase in the critical angle values for films containing more Ni, since Ni has a higher mass density than Al. The mass densities increased from 5.9 g/cm^3^ for HEA2 film up to 8.4 g/cm^3^ for HEN10 film. Also, another trend that was observed was that for the films deposited under N_2_, the critical angle was larger than the values measured for films deposited under vacuum, apart from HEN6 film, which exhibited a lower density that HEA6 film.

The compositions of the synthesized targets and deposited thin films measured by EDS are presented in Table 3. The nickel concentration in experimental alloys was increased from above 9 at. % to more than 30 at. %. This increase in the percentage of nickel led to a gradual reduction in the concentration of other chemical elements.

The results for hardness, nH, and reduced Young’s modulus obtained from nanoindentation measurements are displayed in Table 4. There could be some errors due to the rather limited thickness of the deposited films (around 175 to 200 nm, calculated based on the deposition rate and deposition time), so we tried to use similar penetration depths (hc) for the measurements. One could observe that with the increase in the nickel content, the nanohardness and the reduced Young’s modulus, E_r_, values of the films also increased, which was just the opposite of the behavior observed for bulk alloys (targets). 

The second observation is that the nitride HEA films exhibited higher hardness values than the films deposited under vacuum. We think that these results, obtained using very small indentation depths, were more relevant for these thin films than those reported earlier [27]. Micro-scratch tests with the diamond tip of the nanoindentor were also performed. The films were very adherent, with no cracks or delamination being detected up to a lateral force of 10 mN. The estimated friction coefficients between the diamond tip and the films, which are also displayed in Table 4, were very low.

XPS investigations indicated that the composition of the deposited films on Ti substrates was like that of employed targets and of films deposited on Si and reported in ref. [27]. When comparing the lateral homogeneity of the composition, it was observed that PLD-grown films, regardless of the substrate used, were better than the targets. Since the deposition rate was rather low, the laser beam had enough time to scan a large portion of the target surface for a 0.1 nm increase in the thickness of the deposited film; therefore, the film’s composition corresponded to the average target composition on the laser track and it was very uniform. XPS investigations also revealed that the deposited films contained less than 5–8 at.% oxygen, while those deposited under N_2_ contained several percentages of N atoms bonded in metallic nitride compounds.

OCP represents a parameter that varies due to instability at the film surface–solution interface, so therefore it indicates the corrosion tendency of the investigated material. If the OCP value increases with time, then a passive layer will form on the film surface. This growth is attributed to the passive layer thickening that becomes an even better protector, giving the film anti-corrosive properties. As one can observe in Table 5, all investigated films have the tendency to passivate when immersed in Ringer’s solution, with the highest increase being observed for the thin HEN6 film. The pure Ti sample has an insignificant variation due to the presence on its surface of a high-quality passive layer of titanium dioxide, TiO_2_, which is protective and inert in contact with Ringer’s solution.

**Figure 5:**
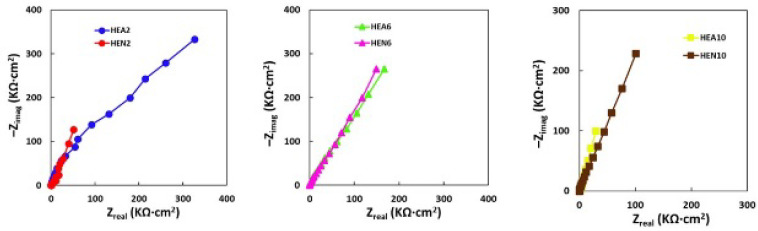
Nyquist plots of thin films: HEA2 and HEN2 (Ni molar ratio x = 0.4), HEA6 and HEN6 (Ni molar ratio x = 1.2), and of HEA10 and HEN10 films (Ni molar ratio x = 2.0).

The repassivation potential represents the potential at which the passive layer is again intact and the highest values, close to that of Ti, as one can observe in Table 6, was measured for the thin HEN6 film. This means that among all investigated samples this one had the largest stability domain. Such layers are not just a chemical barrier, but also a physical one in the corrosion process. For all samples, a near-capacitive response was detected and characterized in Nyquist plots, as one can see for instance on each row of Figure 5 below.

**Figure 6:**
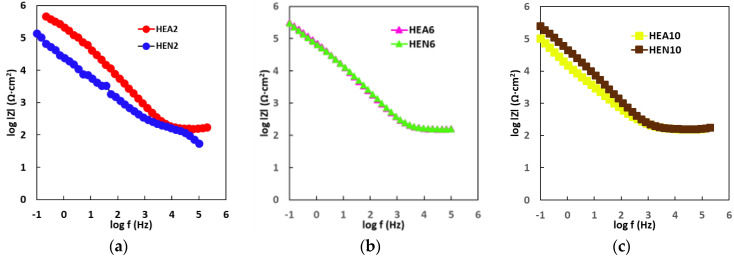
Bode-|Z| plotted data of (**a**) thin HEA2 and HEN2 (of Ni molar ratio x = 0.4), of (**b**) thin HEA6 and HEN6 (of Ni molar ratio x = 1.2), and of (**c**) thin HEA10 and HEN10 films (of Ni molar ratio x = 2.0).

**Figure 7:**
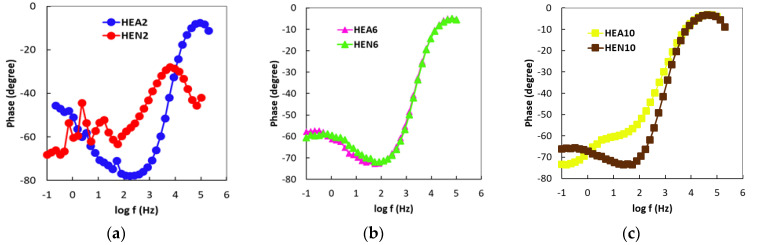
Bode-phase plots of thin films: (**a**) HEA2 and HEN2 (Ni molar ratio x = 0.4), of (**b**) HEA6 and HEN6 (Ni molar ratio x = 1.2), and of (**c**) HEA10 and HEN10 (Ni molar ratio x = 2.0).

**Figure 8:**
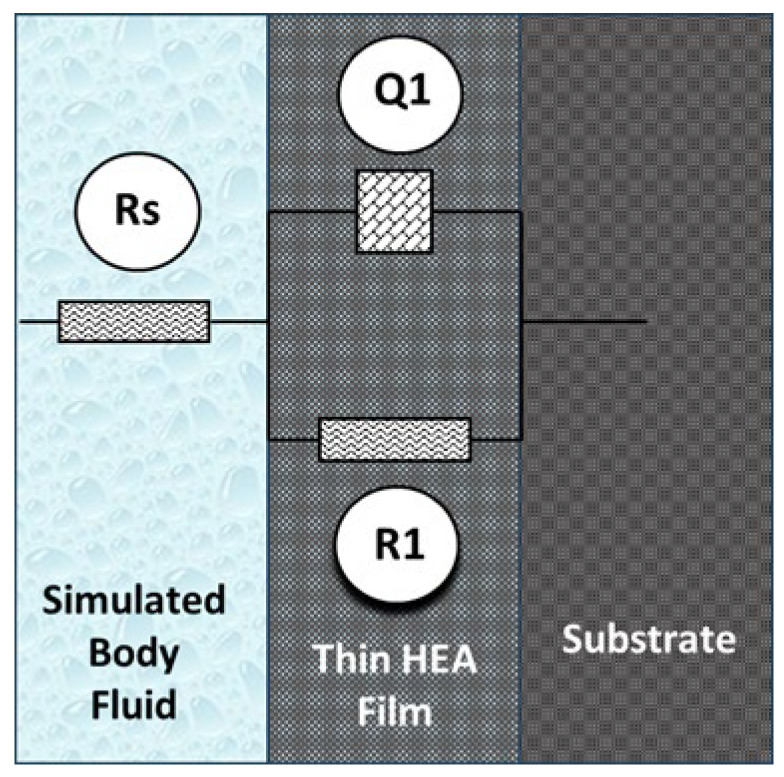
Equivalent circuit used to fit the experimental data.

As one can observe from Figure 6, where Bode- |Z| plots are displayed, for high frequencies almost constant values (horizontal line) of log |Z| versus log (f) were measured, with a phase angle approaching 0° (this is the response of simulated body fluid impedance) in the higher frequency band (1–100 kHz). From the same figure, it appears that the Bode-phase plots show a linear slope the Bode-phase plots of about −1 in log |Z| as the frequency diminishes; meanwhile, the phase angle values approach 80° in the wide low- and mid-frequency range (see Figure 7). Note that this is the expected characteristic behavior of a compact passive film capacitor. The equivalent circuit illustrated in Figure 8 represents the simplest circuit that can be used to fit the experimental data, considering only the charge transfer process. The following components are included in the circuit that has been proposed:

- R1 represents the resistance of the electrolyte, and the value of this resistance can be determined by sweeping at high frequencies.
- The charge transfer resistance component, Rct, is denoted by the letter R2.
- It is possible to establish a connection between the interactions that take place at the electrode/electrolyte interface and the capacitance of the double layer Cdl called Q1. In order to take into account the heterogeneities of the passivated surface, a constant phase element (CPE) has been selected as an alternative to an ideal capacitance.

After fitting the experimental data with the equivalent circuit and comparing the values of R2, as shown in Table 6 for all samples, it can be concluded that the lowest resistance is found for HEN10 and HEN2. For the rest of the samples, the corrosion resistance was higher than that of pure Ti. From all films tested, HEN6 exhibited the best corrosion properties. It is worth mentioning that this film also exhibited the highest N concentration [35,36] and the smallest grain size for both bcc and fcc phases.

## 4. Conclusions

High-entropy alloys from the AlCoCrFeNi_x_ system, with Ni molar ratio x equal to 0.4, 1.2, and 2.0, respectively, were obtained by melting under a protective atmosphere of high-purity argon, with minimal losses of the constituent chemical elements during the technological process. By increasing the nickel content, a change in the microstructure, from pure bcc to a mixture of bcc + fcc phases and finally to a pure fcc structure, was observed. These HEA materials were used as targets in a PLD system to grow thin films, either under high vacuum or a N_2_ atmosphere on Si and highly polished Ti substrates. The deposited films generally reproduced the targets’ chemical composition quite well, but not their phase structure. Films deposited under nitrogen incorporated nitrogen atoms in their lattice, bonded to metal atoms in nitride type compounds, and exhibited a higher density, higher hardness, and better electrochemical properties than the films deposited under high vacuum.

The measured OCP values increased over time for all films, implying that a passive layer was forming on their surface when immersed in SBF solution, with the HEN6 film exhibiting the highest increase. The highest repassivation potential was exhibited by the same film. Impedance measurements indicated high corrosion resistance values for HEA2, HEA6, and HEN6 samples. Overall, HEN6 films, which showed the smallest grain sizes for both phases and the highest N content, exhibited the best corrosion behavior among the investigated films [35,36]. It was noticed that for 24 h of immersion in SBF solution, this film was also a physical barrier to the corrosion process, not only a chemical one. It can be concluded that PLD is an excellent technique to obtain thin HEA or HEN films starting from HEA-synthesized samples, especially when the synthesized alloys used as targets have rather small dimensions.

## Figures and Tables

**Table 1:** Materials, mass, and efficiency of the melting process for HEA targets synthesis.

Target Label	Individual Mass of Chemical Elements, g					Raw Material Mass, g	Batch Mass, g	Efficiency, %
	Al	Cr	Fe	Co	Ni			
HEA2T	4.36	8.36	9.00	9.49	3.79	35	34.88	99.65
HEA6T	3.57	6.87	7.40	7.80	9.36	35	34.74	99.25
HEA10T	3.03	5.83	6.28	6.62	13.24	35	34.92	99.77

**Table 2:** Summary of structural parameter values determined for targets and the thin films grown.

Targets and Thin Films	Position [2θ°]	Grain Size [Å]	Lattice Parameters [Å]	Mass Density [g/cm^3^]
HEA2T	44.473 (bcc)	90 (bcc)	2.879 (bcc)	
HEA2	43.301 (fcc)	67 (fcc)	3.616 (fcc)	5.9
	44.484 (bcc)	123 (bcc)	2.878 (bcc)	
HEN2	44.493 (bcc)	106 (bcc)	2.877 (bcc)	6.0
HEA6T	43.549 (fcc)	112 (fcc)	3.597 (fcc)	
	44.583 (bcc)	114 (bcc)	2.879 (bcc)	
HEA6	43.245 (fcc)	66 (fcc)	3.621 (fcc)	7.4
	44.601 (bcc)	165 (bcc)	2.863 (bcc)	
HEN6	42.897 (fcc)	54 (fcc)	3.649 (fcc)	7.2
	44.628 (bcc)	48 (bcc)	2.869 (bcc)	
HEA10T	43.574 (fcc)	117 (fcc)	3.595 (fcc)	
HEA10	44.723 (bcc)	335 (bcc)	2.863 (bcc)	8.3
HEN10	43.315 (fcc)	45 (fcc)	3.615 (fcc)	8.4
	44.716 (bcc)	253 (bcc)	2.863 (bcc)	

**Table 3:** Atomic percentage composition of the targets and deposited films on Ti substrates.

Element	HEA2T	HEA2	HEN2	HEA6T	HEA6	HEN6	HEA10T	HEA10	HEN10
Al	23.5	22.5	27.0	20.2	20.9	19.1	18.6	18.2	17.9
Cr	23.0	20.3	19.2	20.3	17.1	16.7	17.2	14.3	14.8
Fe	22.6	22.5	20.1	19.2	17.5	15.7	16.8	17.9	16.1
Co	21.7	24.7	20.2	18.1	20.2	15.3	16.9	17.2	16.4
Ni	9.2	10.0	7.5	22.2	24.3	20.2	30.5	32.4	29.8
N	0	0	6.0	0	0	13.0	0	0	5.0

**Table 4:** Summary of mechanical parameter values determined for deposited thin films.

Thin HEA and HEN Films	Indentation Depth, hc, [nm]	Nanohardness, nH, [GPa]	Reduced Young’s Modulus, Er, [GPa]	Friction Coefficient
HEA2	20.6	7.2	79.21	0.1
	34.1	6.8	69.70	
	40.9	6.7	67.83	
HEN2	25.5	9.4	90.39	0.1
	38.2	8.7	79.99	
	42.7	8.6	74.82	
HEA6	21.4	9.5	90.83	0.1
	33.8	9.4	89.40	
HEN6	23.3	13.6	160.48	0.1
	35.3	12.1	157.86	
HEA10	22.8	15.8	187.15	0.09
	31.0	15.1	154.61	
	41.3	14.7	139.72	
HEN10	25.1	16.2	195.77	0.09
	34.7	15.8	171.05	
	46.6	15.3	165.50	

**Table 5:** Open circuit potential of the analyzed samples (initial and after 24 h of immersion in Ringer’s solution) and their repassivation potential.

Sample	E_corr_ Initial [mV]	E_corr_ after 24 h [mV]	E_rp_ [mV]
HEN2	−561	−288	585
HEA2	−268	−182	602
HEN6	−665	−153	780
HEA6	−304	−226	710
HEA10	−293	−162	687
HEN10	−504	−350	401
Ti substrate	−533	−548	-

**Table 6:** Charge transfer resistance of deposited thin films.

Sample	Z [KOhm·cm^2^]
HEN2	24
HEA2	676
HEN6	309
HEA6	316
HEA10	245
HEN10	15
Ti	132

## Data Availability

Data are contained within the article.
